# Dynamic recruitment of ubiquitin to mutant huntingtin inclusion bodies

**DOI:** 10.1038/s41598-018-19538-0

**Published:** 2018-01-23

**Authors:** Katrin Juenemann, Anne H. P. Jansen, Luigi van Riel, Remco Merkx, Monique P. C. Mulder, Heeseon An, Alexander Statsyuk, Janine Kirstein, Huib Ovaa, Eric A. Reits

**Affiliations:** 1 Department of Medical Biology, Academic Medical Center, 1105 AZ Amsterdam, The Netherlands; 2 Leibniz-Forschungsinstitut für Molekulare Pharmakologie, 13125 Berlin, Germany; 3 Netherlands Cancer Institute, 1066 CX Amsterdam, The Netherlands; 4 Department of Chemical Immunology, Leiden University Medical Center, 2333 ZC Leiden, The Netherlands; 5 Department of Cell Biology, Harvard Medical School, Boston, MA 02115 USA; 6 Department of Pharmacological and Pharmaceutical Sciences, University of Houston, Houston, Texas, TX 77204 USA

## Abstract

Many neurodegenerative diseases, such as Huntington’s disease, are hallmarked by the formation of intracellular inclusion bodies (IBs) that are decorated with ubiquitin, proteasomes and chaperones. The apparent enrichment of ubiquitin and components involved in protein quality control at IBs suggests local ubiquitin-dependent enzymatic activity. In this study, we examine recruitment of ubiquitin to IBs of polyglutamine-expanded huntingtin fragments (mHtt) by using synthesized TAMRA-labeled ubiquitin moieties. We show that intracellular TAMRA-ubiquitin is dynamic at mHtt IBs and is incorporated into poly-ubiquitin chains of intracellular substrates, such as mHtt, in a conjugation-dependent manner. Furthermore, we report that mHtt IBs recruit catalytically active enzymes involved in (de)-ubiquitination processes based on novel activity-based probes. However, we also find that the overexpression of the GFP-ubiquitin reporter, unlike the endogenous ubiquitin and TAMRA-ubiquitin, becomes irreversibly sequestered as a ring-like structure around the mHtt IBs, suggesting a methodical disadvantage of GFP-tagged ubiquitin. Our data provide supportive evidence for dynamic recruitment of ubiquitin and ubiquitin (de)-conjugating activity at mHtt initiated IBs.

## Introduction

Targeting and degradation of misfolded proteins is key to cellular health and functioning, as accumulation of misfolded proteins can lead to aggregation and the formation of inclusion bodies (IBs). While many neurodegenerative diseases including Huntington’s disease (HD) are characterized by IBs, it is debated whether these structures represent the actual toxic species. Recently, it was shown that the inclusion body assembly deactivated a risk of apoptosis triggered by soluble mutant Huntingtin (mHtt) and initiated a cellular quiescence that led to a slower death by necrosis^1^.

The recruitment of active 26S proteasomes^2^, ubiquitin (Ub)^3,4^, chaperones but also numerous misfolded proteins suggests that intracellular protein homeostasis is disrupted in HD^5–8^. Although several models have been proposed to explain the ubiquitin proteasome system (UPS) presence in IBs^7,9,10^, the reason for recruitment is still not known.

Ub accumulation at IBs can be found in postmortem human brain material, cell culture and *in vivo* models of HD^4,11^. HD is caused by a CAG repeat expansion in the *Htt* gene, leading to the synthesis of Htt with an extended polyglutamine stretch. A neuropathological hallmark of HD is the presence of Ub-positive IBs composed of mHtt N-terminal fragments containing the polyglutamine stretch^12–14^. Previously, we have shown that aggregated mHtt N-terminal fragments are polyubiquitinated at its N-terminal region, suggesting Ub conjugation at IBs when mHtt is sequestered^15^. This is in agreement with another study showing that only a small percentage of soluble mHtt is indeed ubiquitinated^5^. However, soluble mHtt has a long half-life, indicating that mHtt is not efficiently targeted to the proteasome, leading to intracellular aggregation and IB formation by the intrinsically disordered structure^16,17^. Yet, accumulation of polyubiquitinated material, and UPS substrate reporters have been found in HD mouse models and postmortem human brain material, suggesting a link between the Ub system and the accumulation of mHtt^4^. One explanation of this accumulation could be an overload of the global protein folding capacity by substrate competition for the available chaperones and proteasomes, which in turn leads to disturbances in Ub homeostasis when mHtt is expressed^5,6,18^. This process occurs during intracellular accumulation of mHtt before IB formation and is accompanied by a delayed recruitment of GFP-tagged Ub to IBs^5^. Delayed ubiquitination of IBs was also observed in a HD mouse and *C. elegans* model, implying that formation of IBs and Ub recruitment are two independent processes happening one after another and not at the same time^19,20^. This model is supported by a recent study showing that ubiquitination of destabilized proteins is not required for these proteins to be sequestered into IBs^7^. However, the presence and pattern of Ub at IBs is not well understood and the mechanism underlying the recruitment and dynamics of Ub at already formed mHtt IBs remains unclear.

Here, we used Tetramethylrhodamine-labeled Ub (TAMRA-Ub) to investigate the dynamics of ubiquitination of mHtt IBs in living cells. We show that intracellular TAMRA-Ub behaves like endogenous Ub and is recruited to IBs formed by mHtt. TAMRA-Ub is dynamic and covalently bound to substrates at IBs in a conjugation-dependent manner. Ub recruitment at IBs is, however, not dependent on a preceding ubiquitination of the aggregating protein mHtt, as shown using lysine-dead mHtt that cannot be ubiquitinated but forms aggregates. Our data also show that IBs sequester catalytically active enzymes from the (de)-ubiquitination cascade. Furthermore, in contrast to TAMRA-Ub and endogenous Ub, overexpression of GFP-Ub does not exhibit the same intracellular behavior and is therefore not a suitable tool to study ubiquitination of IBs. This work contributes to a better understanding of intracellular Ub recruitment and dynamics at IBs by the development and usage of small fluorescently labeled Ub moieties.

## Results

### Fluorescent TAMRA-Ub behaves like endogenous Ub

To gain more insight into the dynamics of Ub, synthetic Ub labeled at the N-terminus with TAMRA (TAMRA-Ub) was introduced into living cells by electroporation in order to compare its cellular distribution and incorporation to endogenous Ub.

**Figure 1:**
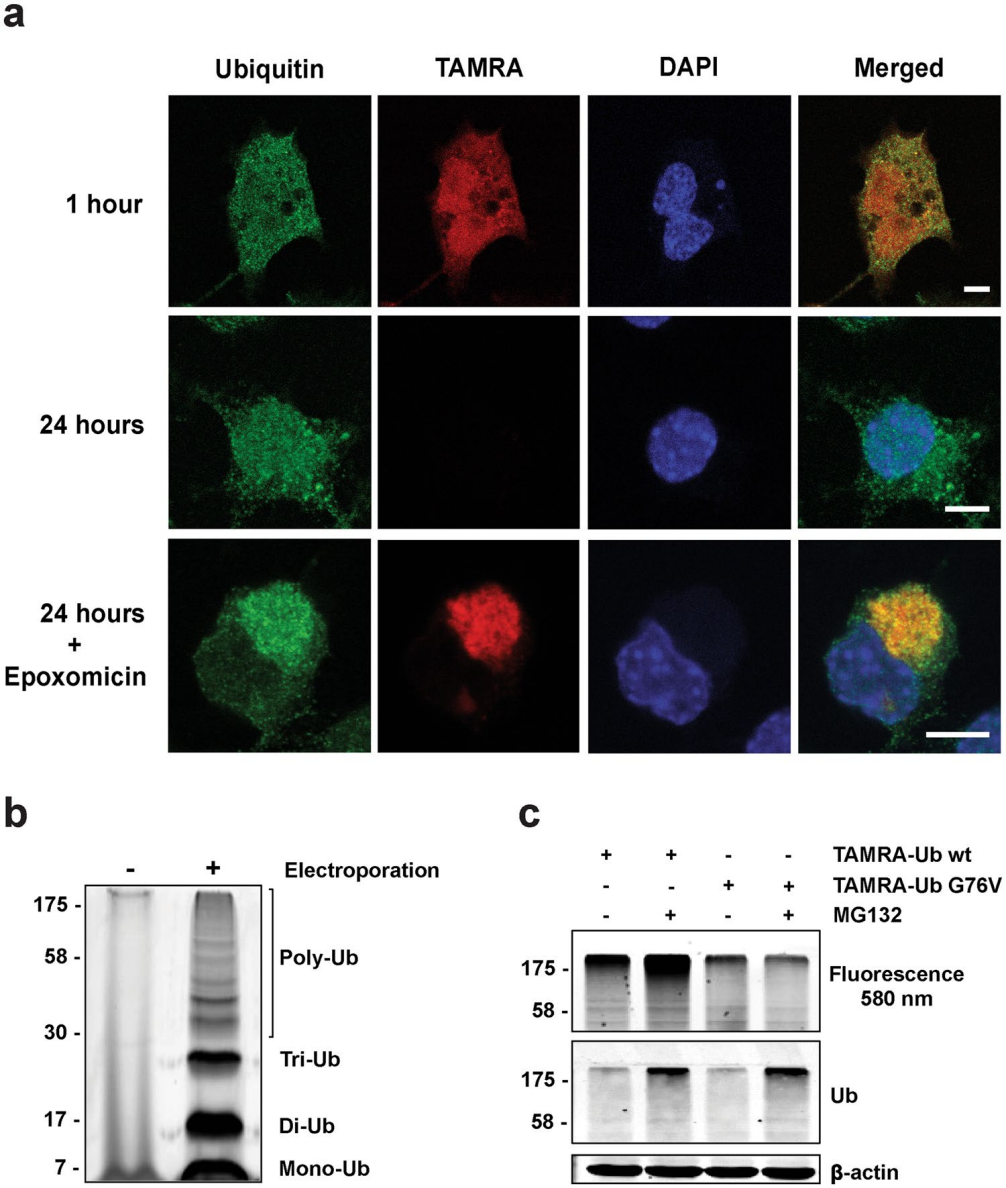
TAMRA-Ub behaves like endogenous Ub. (**a**) Confocal images of intracellular ubiquitin localization in Neuro-2A cells one and 24 hours after TAMRA-Ub electroporation. One hour after electroporation cells were treated with 50 nM epoxomicin and stained with an anti-Ub antibody after fixation. Nucleus was stained with DAPI. Scale bar: 5 µm. (**b**) Neuro-2A cell lysates were harvested two hours after electroporation of TAMRA-Ub and loaded on a SDS-PAGE gel. Fluorescent scan shows TAMRA-Ub incorporated in the poly-Ub tree. (**c**) Fluorescence scan and immunoblot of SDS-PAGE loaded with cell lysates of Neuro-2A cells electroporated with TAMRA-Ub wildtype (wt) or mutant TAMRA-Ub G76V. One hour after electroporation cells were treated with 20 µM MG132 for additional two hours. Ubiquitinated proteins were detected by anti-Ub antibody. β-actin was used as a loading control.

Microscopic analysis of Neuro-2A cells electroporated with TAMRA-Ub showed the intracellular distribution of TAMRA-Ub compared to endogenous Ub, stained with a Ub-specific antibody, one and 24 hours after electroporation (Fig. 1a). One hour after electroporation TAMRA-Ub was present in both the nucleus and cytoplasm, similar to endogenous Ub. However, within 24 hours after electroporation the TAMRA-Ub signal disappeared completely, suggesting degradation of the pool of fluorescent Ub. This is comparable to radioactive-labeled ubiquitin in cells with a half-life of 10–20 hours^21^. In contrast, endogenous Ub signal remains due to constant newly synthesized Ub.

To test whether TAMRA-Ub is targeted for proteasomal degradation and reflects the behavior of endogenous Ub, cells were treated with the proteasome inhibitor Epoxomicin one hour after electroporation. Both TAMRA-Ub and endogenous Ub showed strong perinuclear accumulation after proteasomal inhibition, indicating Ub-enriched aggresome formation. These data are in agreement with previous observations demonstrating Ub-positive aggresome formation after proteasomal inhibition^22,23^.

Previously, it was shown *in vitro* that synthetic Ub can form all Ub-linkage types^24^. To analyze whether TAMRA-Ub is incorporated into endogenous poly-Ub chains, Neuro-2A cells were lysed two hours after electroporation with TAMRA-Ub and loaded on a SDS-PAGE for in-gel fluorescence detection (Fig. 1b). We observed TAMRA-positive ubiquitinated material in the low and high molecular range, suggesting proper incorporation into poly-Ub linkages. Further analysis revealed that TAMRA-Ub incorporation into endogenous polyUb-linked chains is conjugation-dependent as shown by the conjugation-defective mutant TAMRA-Ub G76V containing a glycine to valine mutation at amino acid 76. Treatment of TAMRA-Ub wild type (wt) and the conjugation-deficient mutant (G76V) electroporated Neuro-2A cells with the proteasomal inhibitor MG132 resulted in an increase of high molecular weight TAMRA-positive polyUb material in TAMRA-Ub wt cells only (Fig. 1c, upper panel). TAMRA-Ub G76V was not efficiently incorporated into polyUb chains as shown by Supplementary Fig. S1. To show TAMRA-Ub conjugation onto protein substrates directly, we performed an *in vitro* ubiquitination assay with S5a, a Ub substrate (see Supplementary Fig. S2). Here we could clearly show that TAMRA-Ub like Ub is conjugated as a mono-Ub to the protein S5a, confirming that the TAMRA moiety does not interfere with substrate ubiquitination. Together, this indicates that the N-terminal fluorescent label does not interfere with the Ub-conjugation process and that TAMRA-Ub is covalently bound to ubiquitinated substrates, indicating that TAMRA-Ub behaves like endogenous Ub and is therefore a suitable tool to study intracellular Ub-dependent processes.

### Conjugation-dependent recruitment of TAMRA-Ub to mHtt IBs

**Figure 2:**
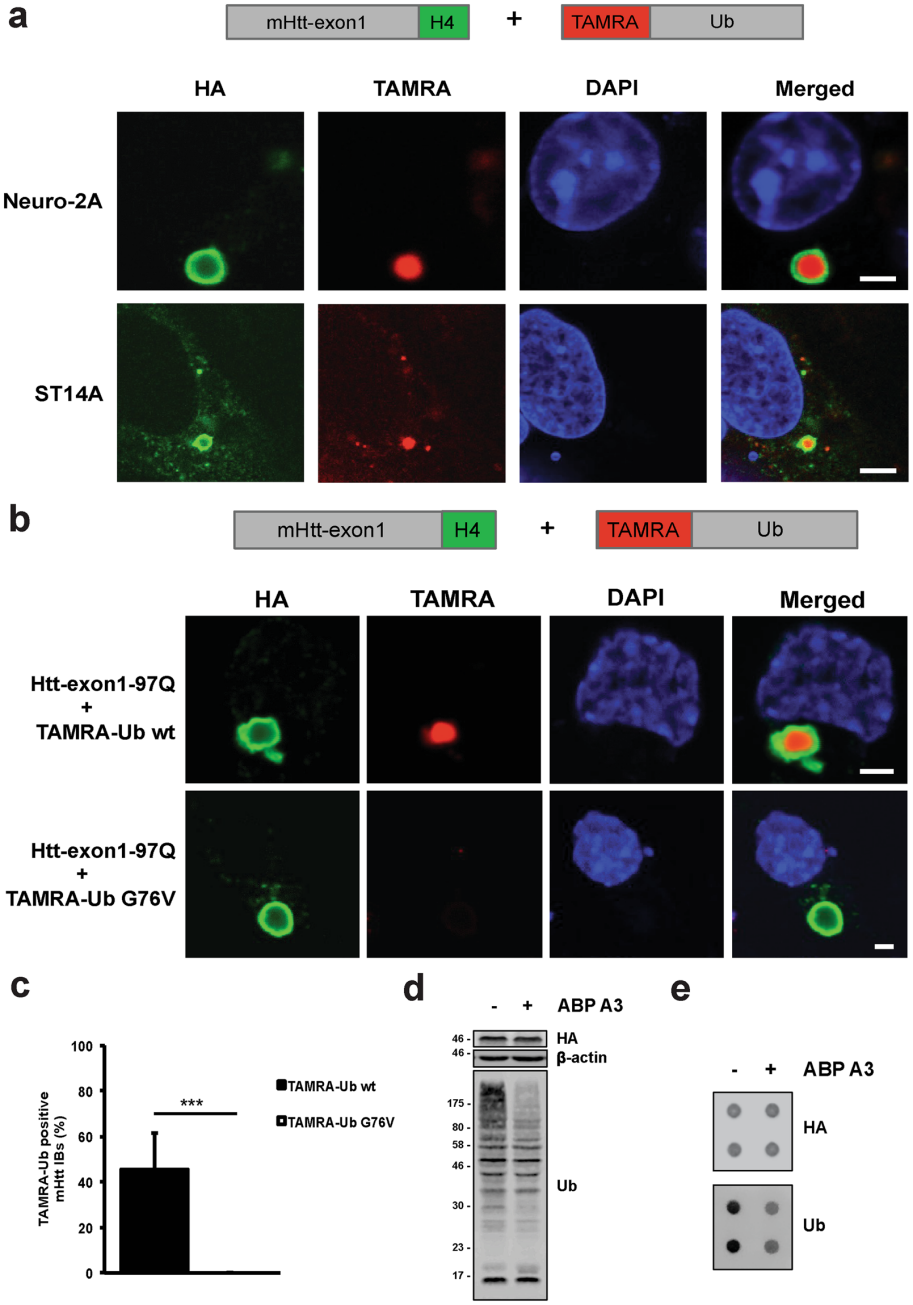
TAMRA-Ub is recruited into Htt IBs. (**a**) Confocal images of Htt-exon1-97Q-H4 protein expressed in transient transfected Neuro-2A cells and stable ST14A cells. 24 hours after transfection cells were electroporated with TAMRA-Ub and incubated for additional 24 hours. Fixed cells were immuno-stained with anti-HA antibody and nuclei were stained with DAPI. Scale bar: 3 µm. (**b**) Confocal images of Neuro-2A cells expressing wild type Htt-exon1-97Q-H4. Cells were electroporated with TAMRA-Ub wild type and the mutant variant G76V 24 hours after transfection and incubated for additional 24 hours. After fixation cells were stained with an anti-HA antibody and the nuclei were stained with DAPI. Scale bar: 2 µm. (**c**) Quantification of TAMRA-Ub positive IBs. Neuro-2A cells expressing Htt-exon1-97Q-C4 were electroporated with TAMRA-Ub wt and the mutant G76V 24 hours after transfection and were incubated for additional 24 hours. Cells were stained with FlAsH and fixed for microscopic analysis. The percentage of TAMRA-Ub and mutant TAMRA-Ub G76V positive Htt IBs was determined ***p < 0.001, (n = 180). Means and SD are shown. (**d**) Western blot of Neuro-2A cells expressing Htt-exon1-97Q-H4 were treated with the E1 inhibitor ABP A3 for 6 hours. Ubiquitinated material was stained with the anti-ubiquitin antibody. β-actin was used as a loading control. (**e**) Filter trap assay (doublets) of mHtt aggregates from Neuro-2A cells transient transfected with the Htt-exon1-97Q-H4 construct for 48 hours. ABP A3 exposure reduces the Ub moiety on SDS-insoluble mHtt aggregates detected by anti-HA and anti-Ub antibodies.

To assess Ub localization at IBs, we studied TAMRA-Ub recruitment to mHtt IBs formed in two different HD cell models. Transiently transfected Neuro-2A cells and stable striatal ST14A cells expressing Htt-exon1-97Q-H4 were electroporated with TAMRA-Ub, and 24 hours later stained with an anti-HA antibody to detect Htt IBs by confocal microscopy (Fig. 2a). In both neuronal cell lines TAMRA-Ub was found to be present in the core of the Htt IBs. To determine the correct optical section of the IB core, sectional profiling of the Z-stack was performed based on the anti-HA antibody staining of the IB. Note that the primary and secondary antibodies are not able to reach the dense core of the Htt IB rather forming a ring around it after fixation, whereas TAMRA-Ub is detectable in the core of the IB. However, when mHtt-transfected Neuro-2A cells were electroporated with TAMRA-Ub and treated with epoxomicin TAMRA-Ub was mainly recruited to aggresomes induced by proteasome inhibition (see Supplementary Fig. S3). In contrast, when in a similar setup no proteasome inhibitor was added but cells were electroporated again with TAMRA-Ub and visualized within 1 hour by microscopy, both free TAMRA-Ub and TAMRA-Ub recruited into IBs was observed. Together, this indicates that in non-stressed cells free TAMRA-Ub is degraded in time, whereas mHtt IBs recruit TAMRA-Ub, which is prevented upon proteasome inhibition where TAMRA-Ub is recruited to epoxomicin-induced aggresomes.

To determine whether the recruitment of TAMRA-Ub to IBs requires covalent binding to substrates, TAMRA-Ub wt and the mutant variant G76V were electroporated into Neuro-2A cells expressing Htt-exon1-97Q-H4. In contrast to TAMRA-Ub wt, the conjugation-deficient mutant G76V was not recruited to IBs, indicating that conjugation is required for recruitment into IBs (Fig. 2b). The loss of diffuse TAMRA-Ub fluorescent signal in the cytoplasm indicates proteasomal degradation over time comparable with TAMRA-Ub wt (Fig. 1a). Quantification of TAMRA-Ub-positive Htt IBs in Neuro-2A cells revealed no TAMRA-Ub G76V co-localization with IBs compared to TAMRA-Ub wt (Fig. 2c).

Moreover, Neuro-2A cells treated with activity-based probe A3 (ABP A3), an E1 inhibitor of ubiquitin and Nedd8 pathways^25^, followed by cell lysis showed efficient inhibition of protein ubiquitination with a decrease of polyubiquitinated material (Fig. 2d). To test whether the inhibition of ubiquitination with ABP A3 reduces the amount of ubiquitinated proteins in the Htt IBs, a Filter trap assay was performed on Neuro-2A cell lysates with IBs formed by the protein Htt-exon1-97Q-H4. Levels of Ub-conjugated proteins in IBs were reduced despite unchanged mHtt levels detected by the HA antibody staining (Fig. 2e). These data indicate that ubiquitination of IBs is dependent on the active Ub-conjugation process rather than sequestration of non-conjugated Ub.

### Recruitment of TAMRA-Ub to mHtt IBs is independent of mHtt ubiquitination

**Figure 3:**
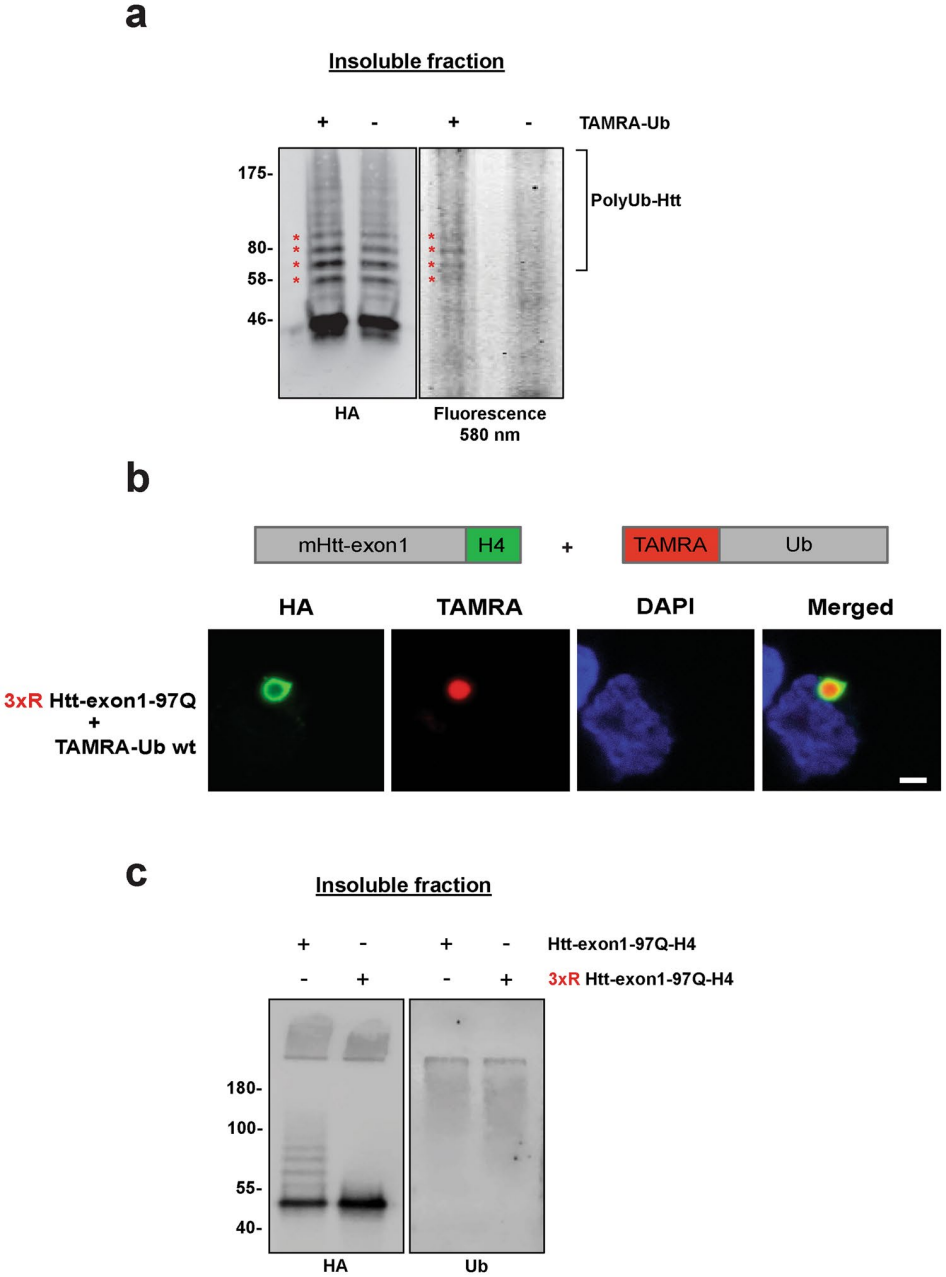
Aggregated mHtt is conjugated with TAMRA-Ub. (**a**) Insoluble fraction of Neuro-2A cell lysate 48 hours after transient transfection with Htt-exon1-97Q-H4. After 24 hours expression cells were electroporated with TAMRA-Ub and incubated for additional 24 hours. Formic acid-dissolved inclusion bodies were loaded on a SDS-PAGE and TAMRA signal of ubiquitinated mHtt (asterisks) was detected by fluorescence scan. mHtt proteins were detected on Western blot by anti-HA immunostaining. Non-electroporated cells expressing Htt-exon1-97Q-H4 were used as a control. (**b**) Confocal images of Neuro-2A cells expressing the mutant 3xR Htt-exon1-97Q-H4. Cells were electroporated with TAMRA-Ub wild type 24 hours after transfection and incubated for additional 24 hours. After fixation cells were stained with an anti-HA antibody and the nuclei were stained with DAPI. Scale bar: 2 µm. (**c**) Insoluble fractionation of Neuro-2A cell lysate 48 hours after transient transfection with Htt-exon1-97Q-H4 or 3XR Htt-exon1-97Q-H4. Formic acid-dissolved inclusion bodies were loaded on a SDS-PAGE. Htt proteins and ubiquitinated material were detected on Western blot by anti-HA and anti-Ub immunostaining.

Next, we investigated the ability of TAMRA-Ub to become conjugated to mHtt in IBs. In a previous study, we could show by using a formic acid-based aggregate solubilization protocol that aggregated mHtt-exon1 is ubiquitinated^15^. Our new data revealed incorporation of TAMRA-Ub into chains of Ub-Htt, shown by in-gel fluorescence of solubilized TAMRA-labeled Htt species (Fig. 3a). TAMRA-Ub Htt species had the same molecular weight as the HA-stained endogenous Ub-Htt conjugates, indicating proper TAMRA-Ub incorporation (asterisks).

To test whether mHtt ubiquitination at IBs is responsible for TAMRA-Ub recruitment to IBs, we expressed the mutant 3xR mHtt-exon1-H4, in which three arginines replace the three N-terminal lysine residues necessary for Htt ubiquitination^26^ and subsequently stained the IBs with an anti-HA antibody (Fig. 3b). TAMRA-Ub was recruited to 3xR mHtt-induced IBs. Furthermore, solubilization of mHtt and 3xR mHtt aggregates reveal Ub-conjugation onto mHtt but not onto 3xR mHtt proteins with similar Ub staining independent of mHtt lysine residues (Fig. 3c), confirming previous data showing that ubiquitination of IBs is also dependent on co-sequestration of other ubiquitinated proteins and not solely on Htt ubiquitination^15^.

Together, these data suggest that TAMRA-Ub behaves like endogenous Ub and is conjugated to the intracellular substrate, mHtt at IBs, however, co-localization of TAMRA-Ub is rather dependent on TAMRA-Ub conjugation to co-sequestered proteins then conjugation to mHtt only.

### TAMRA-Ub is dynamic at mHtt IBs

**Figure 4:**
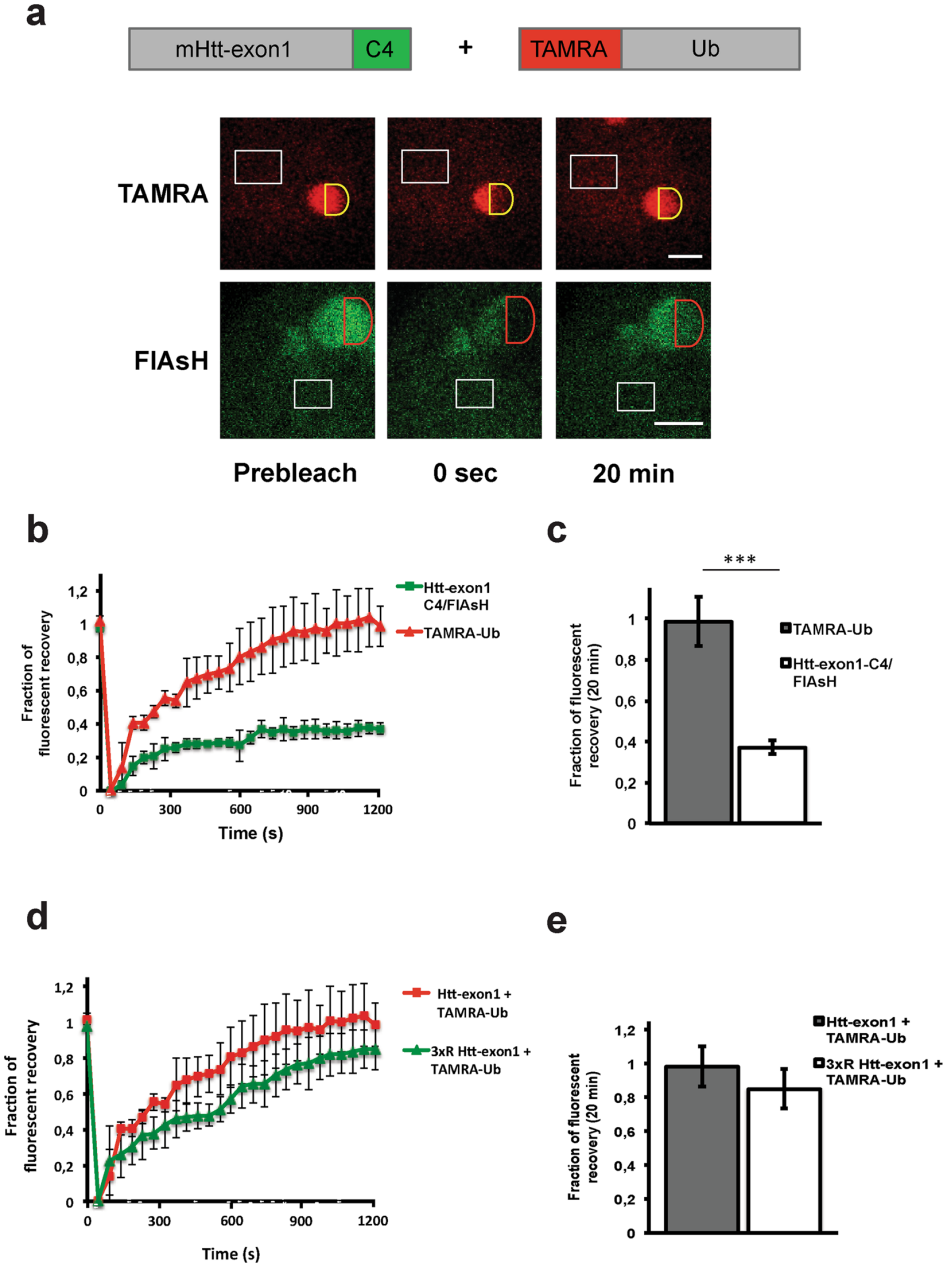
Dynamics of TAMRA-Ub at mHtt IBs. **a)** FRAP analysis of TAMRA-Ub and FlAsH-stained Htt-exon1-97Q-C4 at cytoplasmic IBs of Neuro-2A cells. 24 hours after transfection cells were electroporated with TAMRA-Ub and incubated for additional 24 hours. Red or yellow semi-circles indicate the region-of-interest (ROI) that was photobleached (0 sec). White rectangles indicate non-bleached areas used as a control for photobleaching and normalization. Fluorescence recovery of either mHtt or TAMRA-Ub on cytoplasmic IBs was measured over time. Scale bar: 2 µm. (**b**) Analysis of fluorescent recovery in bleached areas indicates a higher mobility of TAMRA-Ub versus mHtt in IBs over time. (**c**) Quantitative analysis reveals a significantly higher fluorescent recovery of TAMRA-Ub compared to mHtt 20 min after photobleaching. ***p < 0.001. (**d**) Analysis of fluorescent recovery of TAMRA-Ub in bleached areas of mHtt and 3xR mHtt induced cytoplasmic IBs indicates similar ubiquitin mobility. (**e**) Quantitative analysis of TAMRA-Ub on mHtt IBs reveals no significant difference of fluorescent recovery of TAMRA-Ub on mHtt compared to 3xR mHtt 20 min after photobleaching. Means and SD are shown.

The mobility of polyglutamine-expanded proteins, such as 82Q-GFP and Htt-91Q-GFP, localized to the IBs is strongly reduced, showing no on/off rate when performing photobleaching experiments^7,27^. To study dynamics of Ub at mHtt-induced IBs, TAMRA-Ub mobility was determined by FRAP, a technique that measures the mobility of fluorescent molecules in living cells by photobleaching a region of the fluorescent cell and record recovery of fluorescence due to diffusion, active transport, or the on/off rate of fluorescently-tagged proteins from intracellular structures such as an IB. TAMRA-Ub-electroporated Htt-exon1-97Q-C4-expressing Neuro-2A cells were stained with FlAsH to locate TAMRA-Ub-positive Htt IBs in the cytoplasm (Fig. 4a). To measure dynamics of TAMRA-Ub at IBs, recovery of fluorescence was monitored for a time period of 20 minutes after photobleaching. FRAP analysis at IBs clearly showed a significantly higher mobility of TAMRA-Ub compared to FlAsH-stained mHtt-exon1 with a mobile fraction of 99 ± 12% and 37 ± 3.5% (p < 0.001), respectively (Fig. 4b,c).

To test whether TAMRA-Ub mobility at IBs was dependent on Htt ubiquitination, the fluorescent recovery of FlAsH-stained mHtt-exon1 was compared to the ubiquitin conjugation -deficient mutant 3xR mHtt-exon1. We observed no significant difference in the mobile fraction of TAMRA-Ub at IBs formed by mHtt and 3xR mHtt, respectively (Fig. 4d,e). These data confirm our previous results, indicating Ub dynamics independent of Htt ubiquitination on IBs (Fig. 3b).

### Ub is present in the core and periphery of the IBs in HD mouse models

**Figure 5:**
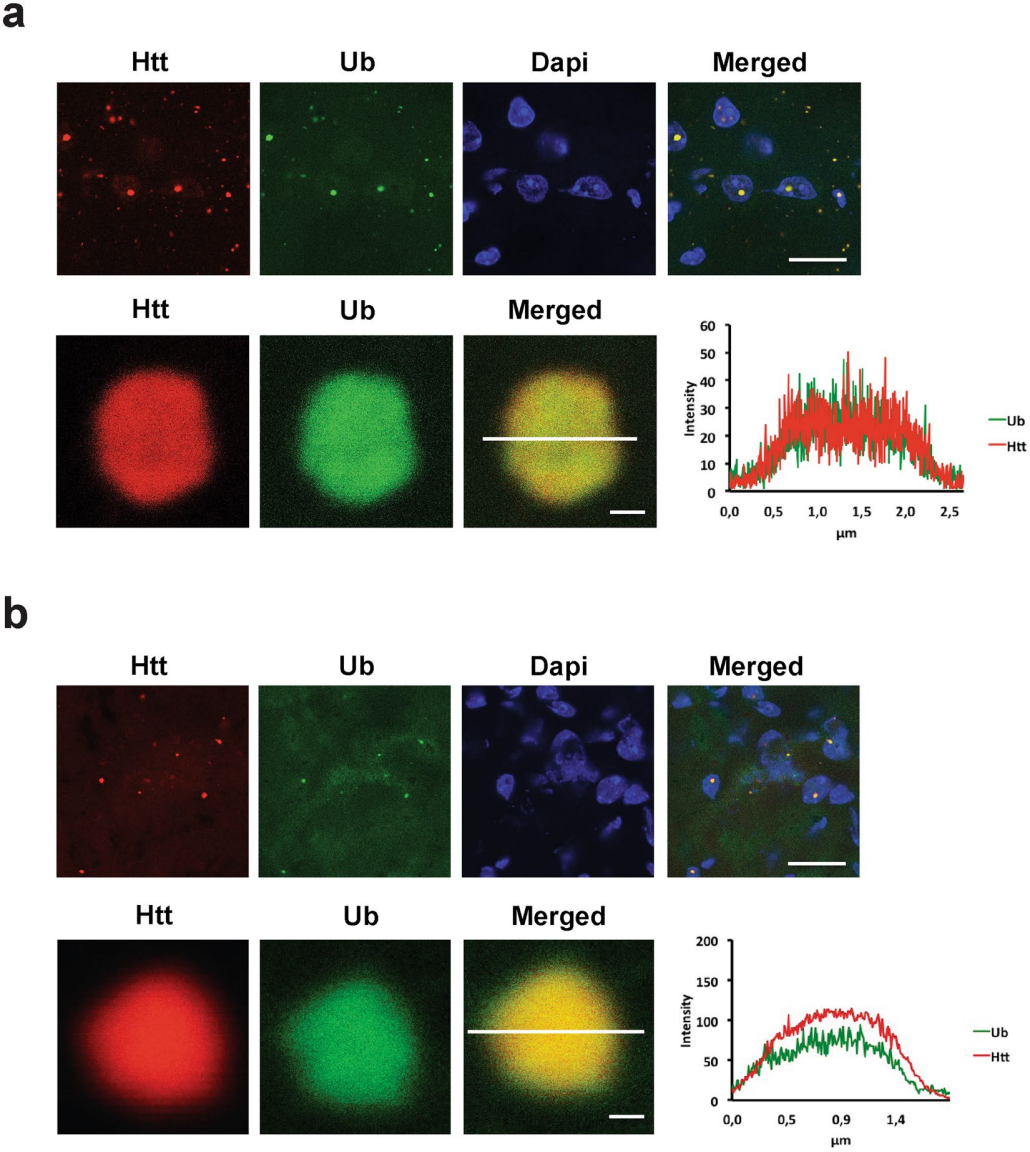
mHtt IBs in transgenic R6/2 and homozygous HdhQ150 mouse models are ubiquitin positive. Confocal images of ubiquitin co-stained with mHtt IBs in (**a**) 14-week-old R6/2 mouse brain and (**b**) 22-month-old HdhQ150 mouse brain slices. Exemplary confocal pictures of mouse cortex tissue containing ubiquitin-positive aggregates, note that ubiquitin antibody staining is present in the core of the aggregate and correlates with the intensity of the Htt antibody staining. IBs were stained with an anti-Ub antibody P4D1 and anti-Htt antibody S829. Nuclei were stained with DAPI. Scale bar upper panel is 8 µm; lower panel is 0.25 µm.

In order to validate our findings *in vivo*, we studied ubiquitination of Htt IBs in two different HD mouse models by immunofluorescence staining of mouse brain sections. Cortex tissue from the transgenic R6/2-HD model, expressing human mHtt-exon1^28^, and the knock-in HdhQ150 model was used^29^. Due to the sectioning of tissues the core of intracellular inclusion bodies was accessible for staining with the S829 antibody that was raised against the N-terminus of polyglutamine-expanded Htt-exon1^30^, in combination with a ubiquitin antibody. In both mouse models, ubiquitin is present in cytoplasmic and nuclear IBs, respectively (Fig. 5a,b). Furthermore, we measured equal fluorescent intensities of both antibody stainings, which overlap in the periphery and core of the IB confirming our cell-based studies with TAMRA-Ub that endogenous Ub is present in the core and periphery of the IBs in HD cell models (Fig. 2a).

### IBs sequester catalytically active enzymes from the (de)-ubiquitination cascade

With the development of activity-based probes and novel inhibitors it has become possible to identify and localize enzymes involved in protein (de)-ubiquitination. The activity-based probe for E1-E2-E3 enzymes (Cy5-Ub-Dha) reacts with the active site cysteine residue, thereby trapping proteins involved in the E1-E2-E3 cascade^31^. Cy5-Ub-PA is a specific inhibitor of all three major DUB (deubiquitinating enzyme) families: UCH, USP and OTU, allowing the fluorescent labeling of intracellular DUBs.

**Figure 6:**
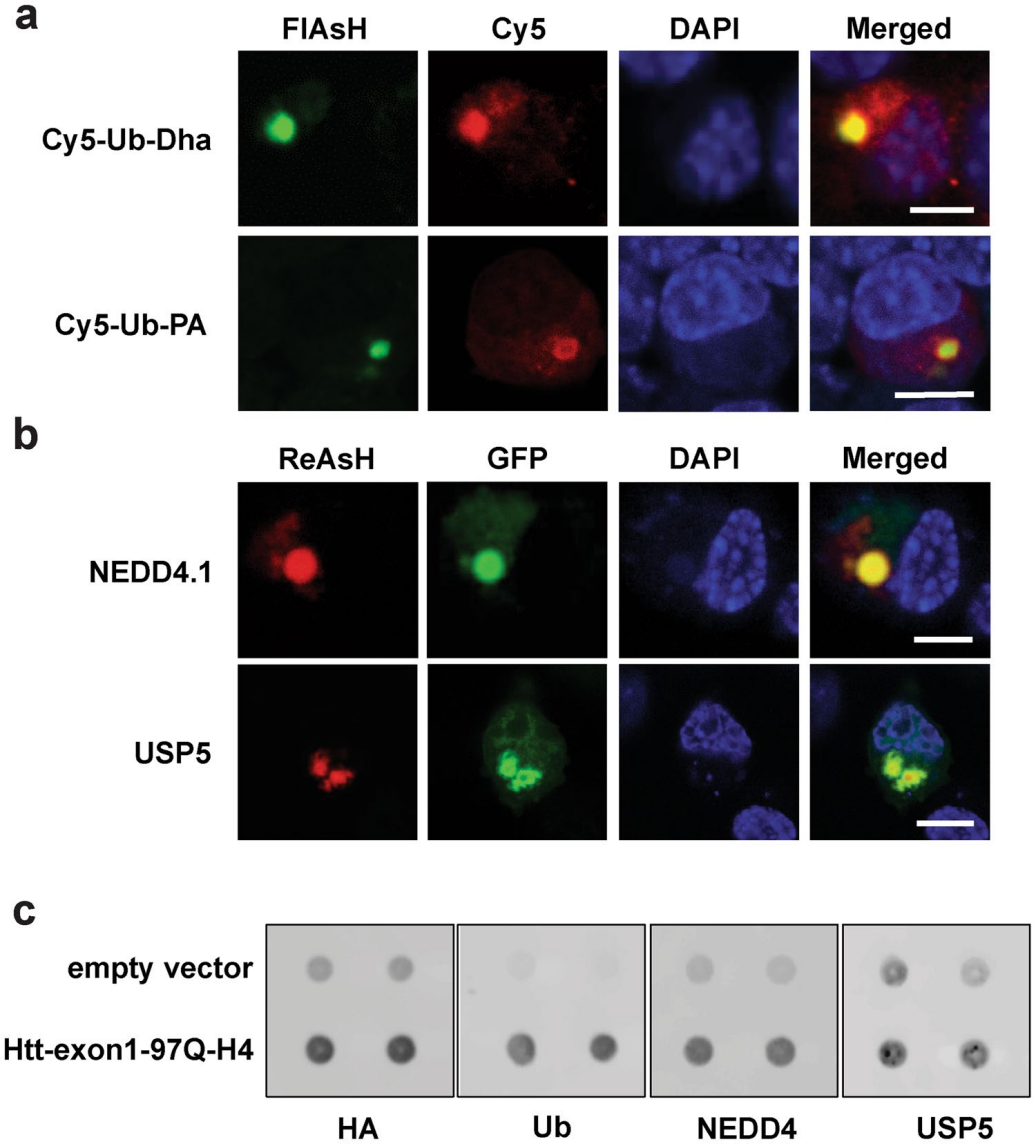
mHtt IBs sequester active enzymes from the (de)-ubiquitination cascade. (**a**) Confocal images of Htt-exon1-97Q-C4 protein expressed in transient transfected Neuro-2A cells. 24 hours after transfection cells were electroporated with the Cy5-labeled activity-based probes Ub-Dha, which labels active E1-2-3 enzymes, and Cy5-Ub-PA, which labels active DUBs. One hour after electroporation cells were FlAsH-stained and fixed for imaging. (**b**) Confocal images of transient transfected Neuro-2A cells co-expressing Htt-exon1-97Q-C4 with GFP-NEDD4.1 and GFP-USP5, respectively. 24 hours after transfection cells were ReAsH-stained and fixed for imaging. Nuclei were stained with DAPI. Scale bar: 6 µm. (**c**) Filter trap assay (doublets) of mHtt aggregates from Neuro-2A cells transient transfected with the Htt-exon1-97Q-H4 construct for 48 hours. mHtt aggregates sequester endogenous NEDD4 and USP5. Aggregates are stained by anti-HA, anti-Ub, anti-NEDD4 and anti-USP5 antibodies.

Previous studies have shown that mHtt IBs are positive for Ub and proteasomes, suggesting UPS-related enzymatic activities within IBs^2,32^. To analyze whether IBs of mHtt-exon1-97Q-C4-transfected Neuro-2A cells recruit active (de)-ubiquitinating enzymes, cells were electroporated with chemically synthesized activity-based probes for the E1-E2-E3 enzyme cascade and DUBs. Confocal microscopy analysis of FlAsH-stained IBs revealed enrichment of catalytically active enzymes of the ubiquitination machinery within IBs (Fig. 6a). Interestingly, the DUB-activity probe labeled active cysteine protease DUBs recruited at IBs in ring-like structure in contrast to the E1-E2-E3 enzymes probe, suggesting that active DUBs are mainly present at the periphery of IBs.

Previously, it was shown that K63-linked polyubiquitin is associated with aggregates, probably playing a role in aggresome formation and/or clearance^33,34^. To further study recruitment of specific enzymes involved in protein (de)-ubiquitination at cytoplasmic IBs the K63-specific E3 ligase NEDD4.1 and the DUB USP5, were co-expressed with the mHtt-exon1 protein. Confocal microscopy analysis clearly showed NEDD4.1 and USP5 co-localization with ReAsH-stained mHtt IBs (Fig. 6b). The co-sequestered DUB USP5 is mainly localized on the periphery of the IB, which is in agreement with the observed DUB activity probe staining of IBs (Fig. 6a). Similarly, endogenous USP5 and NEDD4 are recruited into IBs, as shown by Filter trap assay (Fig. 6c). These results demonstrate that mHtt IBs recruit catalytically active proteins involved in protein (de)-ubiquitination.

### GFP-Ub becomes irreversible sequestered to mHtt IBs

To gain more insight in the dynamics and pattern of substrate ubiquitination, several studies employed Ub N-terminally tagged with a fluorescent fusion protein^5,35,36^. These fusion proteins have been broadly used to study for example the behavior of Ub in context of protein trafficking, DNA repair, degradation and IB formation. Live cell imaging experiments showed that the mobile fraction of diffused GFP-Ub is slower compared to the conjugation-deficient mutant GFP-Ub^K0, G76V^, indicating that the main pool of GFP-Ub is conjugated to substrates^36^.

**Figure 7:**
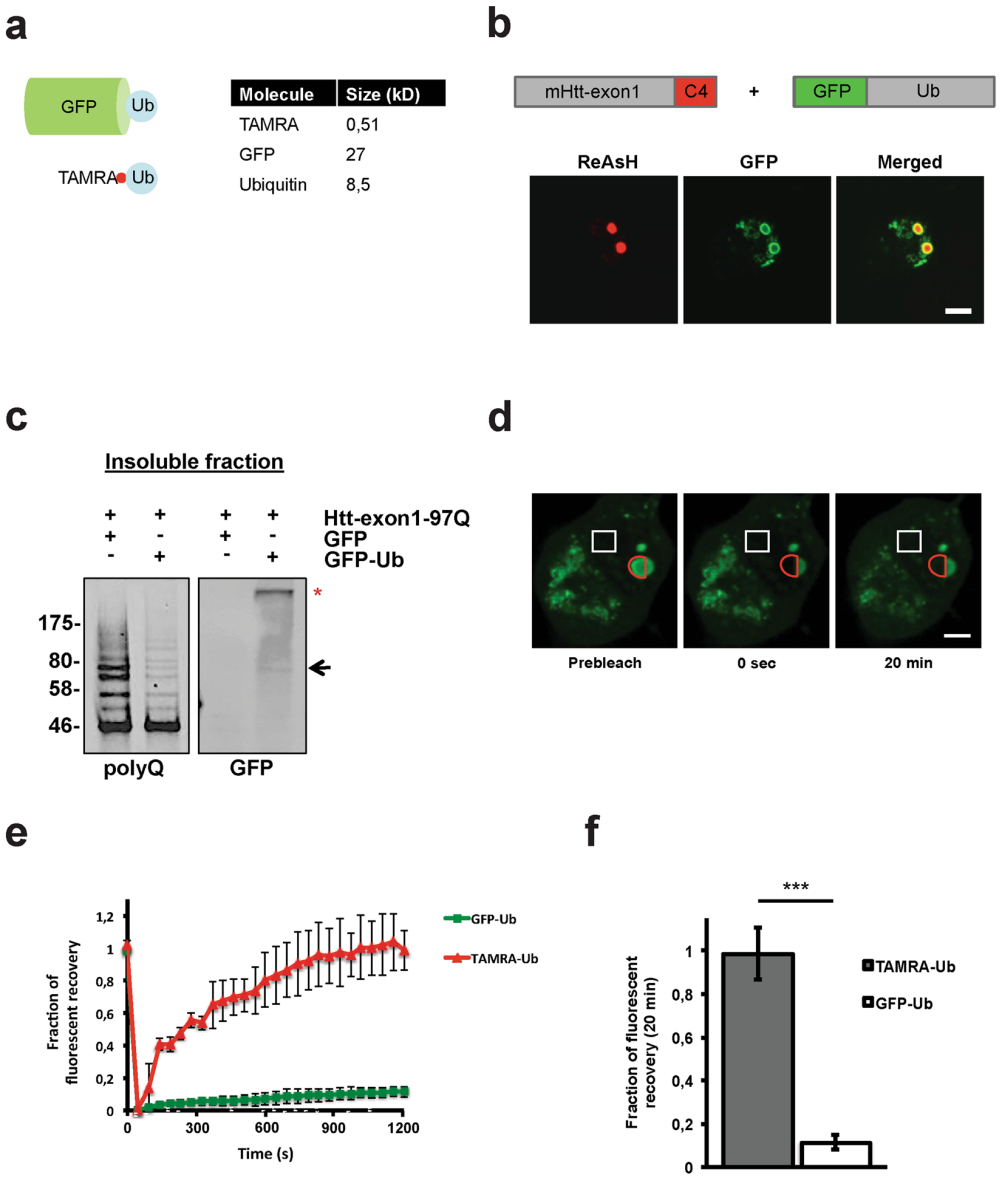
The action of overexpressed GFP-Ub on mHtt IBs is not equivalent to TAMRA-Ub. (**a**) Scheme of GFP-Ub versus TAMRA-Ub. (**b**) Confocal images of transient transfected Neuro-2A cells co-expressing Htt-exon1-97Q-C4 and GFP-Ub. 48 hours after transfection cells were ReAsH-stained and fixed for imaging. Scale bar: 5 µm. (**c**) Insoluble fraction of Neuro-2A cell lysate 48 hours after transient co-transfection of cells with Htt-exon1-97Q and either GFP or GFP-Ub. Formic acid-dissolved aggregates were loaded on a SDS-PAGE and immunoblotted with the antibodies anti-polyQ and anti-GFP. A band of the size of mono-ubiquitinated GFP-Ub-mHtt was detected (arrow) next to GFP-Ub in a high molecular range (asterisk). (**d**) FRAP analysis of GFP-Ub in cytoplasmic IBs of Neuro-2A cells 48 hours after transfection. Fluorescence recovery of GFP-Ub on cytoplasmic Htt IBs was measured over time. Red semi-circle indicates where bleaching was directed. White rectangle indicates non-bleached area used as a control for photobleaching and normalization. Scale bar: 5 µm. (**e**) Analysis of fluorescent recovery in bleached areas of Htt IBs indicates a higher mobility of TAMRA-Ub versus GFP-Ub over time. (**f**) Quantitative analysis reveals a significantly higher fluorescent recovery of TAMRA-Ub compared to GFP-Ub 20 min after photobleaching. ***p < 0.001. Means and SD are shown.

The fluorescent protein GFP, with a size of 27 kDa, is approximately three times larger than the 8.5 kDa protein Ub, which could interfere with endogenous substrate ubiquitination or Ub chain formation (Fig. 7a). When Neuro-2A cells were transiently co-transfected with plasmids encoding GFP-Ub and Htt-exon1-97Q-C4, GFP-Ub formed a distinct ring around the mHtt IBs labeled by ReAsH with overlapping fluorescent signals in the periphery of the IB (Fig. 7b). This is in contrast to both TAMRA-Ub and endogenous Ub distribution in IBs *in vitro* and *in vivo*, respectively, where Ub can be found in both the core and periphery of the IBs (Fig. 2a,b and 5a,b). To test whether the formation of a GFP-Ub ring is due to expression time needed by the transiently co-transfected GFP-Ub plasmids, resulting in delayed recruitment to only the outer layer of mHtt, we transfected Neuro-2A cells with GFP-Ub first, followed by transfection with Htt-exon1-97Q-C4 24 hours later, and vice versa (Supplementary Fig. S4). In both cases Htt IBs exhibited similar GFP-Ub rings independent of a pre-existing GFP-Ub pool at the time of IB formation. In addition, co-transfection with different plasmid ratios of mHtt versus GFP-Ub was performed to analyze whether GFP-Ub locatization depends on the intracellular level of GFP-Ub (see Supplementary Fig. S5). Since no differences in Htt IBs recruitment of GFP-Ub in a ring-like structure was observed, this indicates that the typical recruitment of GFP-Ub is not due to the levels or the timing of GFP-Ub expression.

To assess whether expression of GFP-Ub affects the endogenous ubiquitination of the substrate mHtt, Htt-exon1-97Q was co-expressed with GFP-Ub and GFP as control, respectively. After 48 hours cells were lysed and the IB containing insoluble fraction was isolated and dissolved with formic acid. In contrast to mHtt co-expressed with the GFP control, GFP-Ub co-expression lead to a decrease in mHtt ubiquitination in the low molecular range shown by the immunostaining with the antibody against the polyglutamine-stretch (Fig. 7c). Conjugation of GFP-Ub onto mHtt was shown with a GFP antibody (arrow). Moreover, GFP-Ub conjugates (asterisk) were detectable in the insoluble fraction of Neuro-2A cells expressing mHtt, which might represent GFP-Ub incorporation into polyUb chains of mHtt and other sequestered proteins. Further analysis concerning the dynamics of GFP-Ub at IBs by FRAP revealed that GFP-Ub is immobile compared to TAMRA-Ub with a mobile fraction of 11 ± 3.2% (p < 0.001), suggesting sequestration of GFP-Ub in a ring-like structure at mHtt IBs with no on/off rate in time (Fig. 7e,f), similar as what we observed before^4^.

These data indicate that the intracellular action of the fusion protein GFP-Ub on mHtt IBs is not equivalent to endogenous Ub or electroporated TAMRA-Ub. GFP-Ub becomes irreversibly sequestered around the IB, which is in contrast to our previous results *in vivo*. This indicates that overexpression of Ub N-terminally tagged with a fluorescent fusion protein is not a suitable tool to study intracellular Ub conjugation and dynamics at IBs and obtained data need to be considered carefully.

## Discussion

HD is a well-studied polyglutamine disease and an ideal model to investigate the composition and dynamics of IBs formed by aggregation-prone N-terminal fragments of mHtt and co-sequestered proteins. The presence of Ub, active proteasomes, chaperones and sequestered proteins that are in a nonnative state at IBs, indicates that intracellular protein homeostasis is disrupted^2,5–8,11^. However, the mechanism underlying the recruitment and dynamics of Ub at already formed mHtt IBs remains unclear.

In the present study, we have shown that TAMRA-Ub, a newly developed fluorescently labeled tool to study intracellular Ub kinetics, is dynamic at mHtt IBs and is recruited in a conjugation-dependent manner. TAMRA-Ub is a synthetic Ub derivative, which is incorporated into endogenous Ub chains and able to form all Ub-linkage types. This makes it a suitable tool for studies to follow intracellular Ub conjugation. Furthermore, TAMRA-Ub is conjugated onto substrates forming mono-ubiquitinated protein species (Supplementary Fig. S2). The advantage of using TAMRA-Ub is the introduction of the synthetic polypeptide to cellular systems for posttranslational protein modification analysis at any desired time point and the small molecular weight of the fluorophore (0.51 kDa) in comparison with the already small Ub protein (8.5 kDa), which should not interfere with its function.

Interestingly, TAMRA-Ub is distributed throughout the entire IB and not in a ring around it. This is in contrast to the ring-like distribution of the chaperone Hsp70 but also proteasomes that were initially within the core of IBs but redistributed towards the periphery in time^2,37^. We confirmed this result with immunostaining of mouse brain sections in two different HD mouse models, where the core of intracellular IBs was accessible for the Ub antibody. These data show that the core of mHtt-induced IB is amongst others composed of ubiquitinated proteins. Both the TAMRA-Ub conjugation-deficient mutant G76V and the treatment of cells with the E1 inhibitor ABP A3 proved that Ub recruitment to IBs is conjugation-dependent, as shown before^7^. In this work, Ub fused to YFP was immobilized when recruited to IBs, with no exchange between IB and the cytosol or within the IB itself. In contrast, our FRAP analysis clearly showed that there is TAMRA-Ub mobility over a time period of 20 minutes, suggesting the reversible recruitment of ubiquitinated proteins, or continuous ongoing (de)-ubiquitination events within the IB, leading to the exchange of Ub itself between the IB and the cytoplasm. In addition, our data show that active DUBs and enzymes from the ubiquitination cascade are co-sequestered at IBs corroborating the possibility that Ub (de)-conjugation occurs at IBs directly. This is in accordance with our previous study, which already showed enzymatic activity at IBs by staining of mHtt IBs in cell culture and the R6/2 mouse model with activity-based probes for catalytic activity of proteasomes^2^. There is no strong evidence yet that recruited 26 S proteasomes associated with polyubiquitinated proteins at IBs are capable of dissociating these aggregated proteins for proteolysis. However, it was recently shown that the proteasome shuttle factor UBQLN2 acts with the HSP70-HSP110 disaggregase machinery to clear protein aggregates via the 26 S proteasome^38^. Alternatively, IBs can be cleared by macroautophagy in a ubiquitin-dependent manner, as lysine 63-linked ubiquitination promotes the formation and autophagic clearance of IBs associated with neurodegenerative diseases. Similar to proteasomal degradation, this should be preceded with a disaggregation event in order to target insoluble mHtt for clearance by autophagy as IBs are too large to be cleared by autophagy^34^.

To differentiate between direct mHtt ubiquitination at IBs and ubiquitination of other sequestered substrates, the conjugation-deficient mutant 3xR mHtt was expressed, showing that IBs formed by this mutant are still also positive for Ub^15,26^. While FRAP analysis of IBs can only measure the entire FlAsH-stained IBs discrimination between mainly immobile mHtt and a fraction of dynamic, soluble mHtt species being recruited, is not possible. However, our data clearly shows that Ub dynamics on IBs formed by mHtt and the 3xR mHtt mutant are comparable.

Soluble mHtt is not efficiently targeted by the 26 S proteasome as apparent by its long half-life, and the lack of efficient ubiquitination for proteasomal degradation leads to intracellular aggregation driven by the intrinsic disordered structure of mHtt^5,15,16^. Other studies investigated the targeting of Htt to the UPS by analyzing Htt tagged with a fluorescent protein or by performing native immunoprecipitations to pulldown ubiquitinated Htt from cells or tissue, however, the influence of the fusion protein on Htt degradation and the co-immunoprecipitated Ub conjugates associated with Htt might lead to misinterpretation of results^39,40^. Previously, we could show that aggregated mHtt is polyubiquitinated at IBs, suggesting that N-terminal mHtt fragments become ubiquitinated at IBs^15,17^. Inhibition of the proteasome did not increase mHtt levels at IBs, suggesting that the ubiquitination linkages are insufficient for proteasomal recognition, or that polyubiquitinated mHtt remains irreversibly sequestered in IBs and that proteasomes are unable to target or extract aggregated mHtt. Both models might explain our findings of the presence of Ub-conjugated mHtt and active enzymes involved in the protein ubiquitination process at IBs. Why DUB activity is mainly present in the periphery and E1-E2-E3 activity throughout the IB, and whether this recruitment is a specific mechanism will be interesting to explore. Co-sequestration of the specific E3 enzymes ITCH, TRAF6, UBE3A, UHRF2 and Parkin with mHtt IBs was shown by fluorescent staining in cellular models of HD^32,40–44^. Whether these proteins are involved in the ubiquitination of mHtt at the IB site is still unknown.

Thus, by investigating the accumulation of Ub-conjugates at IBs, our results show that IBs are far from static structures that irreversibly sequester proteins, as (de)ubiquitination is continuous ongoing. For the first time, we showed that IBs harbor enzymatically active proteins involved in the (de)-ubiquitination process. Further research is needed to elucidate possible mechanisms by which Ub-conjugation of aggregated proteins at IBs might be involved in chaperone-mediated disaggregation by specific intracellular disaggregation complexes in order to target these proteins to ubiquitin-specific clearance pathways synergistically.

## Methods

### Constructs

Wild type and 3xR mutant variant of Htt-exon1-97Q with an H4- (His-HA-HA-His) or C4- (Tetracysteine) tag were previously described^17^. GFP-Nedd4.1 was kindly provided by P. Hordijk (VUMC, Amsterdam, The Netherlands). GFP-USP5 was kindly provided by S. Urbé (University of Liverpool, UK) and GFP-Ub was generated as described previously^3^.

### Generation of ST14A cell line stably expressing mHtt

ST14A cells, previously derived from rat embryonic striatum^45^, were used to generate a stable cell line expressing the N-terminal fragment of mHtt with the H4-tag. Htt-exon1-97Q-H4 was cloned into the pLenti6.3-DEST vector for expression in cells using the ViraPower Lentiviral Expression Systems (Invitrogen). Transduction was performed by infection with viral supernatant from the 293FT producer cell line. Blasticidin (10 μg/ml) was used for selection of stably transduced cells. Single colonies were screened for Htt expression by western blot.

### Cell culture and transfection

Neuro-2A and ST14A cells were maintained in DMEM supplemented with 10% fetal calf serum, 1 mM glutamine, 100 U/ml penicillin, and 100 μg/ml streptomycin in a humidified incubator with 5% atmospheric CO_2_. Neuro-2A cells were cultured at 37 °C and ST14A cells at 33 °C. Cells were seeded in 35 mm^2^ culture dishes and transfected with either Polyethylenimine (Neuro-2A cells) or Lipofectamine 2000 (ST14A cells) according to the manufacturer’s instructions (Polysciences Europe). The E1 activating enzyme activity-based probe (ABP) A3 was a kind gift from A. Statsyuk (Northwestern University) and dissolved in DMSO^46^. Neuro-2A cells were transfected and incubated for 48 hours. ABP A3 was added to the cells for the last 6 hours of incubation with an endconcentration of 250 nM. Treatment with DMSO served as a control.

### Ubiquitin Synthesis

Wild type (wt) and mutant (G76V) TAMRA-ubiquitin (TAMRA-Ub), deubiquitinating enzyme (DUB) activity probe Cy5-Ub-PA and the activity-based E1-E2-E3 probe Cy5-Ub-Dha were synthesized as described previously^24,31,47,48^. Powder of synthesized TAMRA-Ub and DUB activity-based probe were dissolved in 10 μl DMSO then 10 μl ddH_2_O was added and subsequently diluted to 1 ml with mannitol buffer to prepare a 100 μM working solution for cell electroporation. The activity-based probe Cy5-Ub-Dha was first dissolved in 10 μl DMSO and added to 1 ml mannitol buffer to prepare a working solution of 5–50 mg/ml.

### Electroporation

Electroporations were performed in a 35 mm^2^ dish using the Bio-rad Gene pulser II Electroporation machine supplemented with a Bio-rad radio frequency (RF) module and a Petri Pulser electrode (BTX Harvard Apparatus). After removing the medium, cells were washed two times with warm (37 °C) phosphate buffered saline (PBS) followed by two washes with cold (4 °C) mannitol buffer (2 mM HEPES, 15 mM K_2_HPO_4_/KH_2_PO_4_, 250 mM mannitol, 1 mM MgCl_2_, pH = 7.2). After in-dish electroporation, the cells were incubated with the synthetic Ub polypeptides dissolved in mannitol buffer on ice for five minutes, followed by two times washing with cold (4 °C) mannitol buffer and warm (37 °C) PBS. Warm conditioned medium was put back on the cells to increase survival. Due to extreme light sensitivity of the fluorophores, all procedures were performed in the dark. One hour after electroporation, cells were either incubated with 50 nM epoxomicin (Sigma-Aldrich) for additional 23 hours or 20 μM MG132 for one hour followed by either 4% PFA fixation on coverslips for live cell analysis or cell harvest.

### SDS-PAGE and immunoblotting

For SDS-PAGE and subsequent Western blotting cells were harvested in lysis buffer (50 mM Tris/HCl pH 7.4, 150 mM NaCl, 1 mM EDTA, 1% Triton-X100, 20 mM NEM, supplemented with complete mini protease inhibitor cocktail (Roche)). After protein concentration was determined with a Bradford protein assay, total cell lysates were boiled for 10 min at 99 °C with 1× laemmli sample loading buffer and loaded onto a SDS-PAGE gel. TAMRA-Ub was detected with in gel fluorescence using a Typhoon imager (GE Healthcare) with the 580 BP 30 filter. For immunoblotting proteins were transferred to a nitrocellulose membrane (0.45 µm pore size, Schleicher & Schuell) and blocked with 5% milk, incubated with primary antibodies anti-polyQ (1:1000, Sigma-Aldrich 3B5H10), anti-HA (1:1000, Sigma- Aldrich, H3663), polyclonal rabbit anti-GFP (1:1000, kindly provided by J. Neefjes, NKI, The Netherlands), anti-β-actin (1:1000, Santa Cruz, SC-130656) and anti-ubiquitin (1:100, Sigma-Aldrich, U5379), and subsequently incubated with secondary antibodies IRDye 680 or IRDye 800 (1:10,000; LI-COR Biosciences). Infrared signal was detected using the Odyssey imaging system (Licor). For Soluble/insoluble fractionation and Filter trap assay cells were harvested and lysed according to the protocol described before^15^. For the Filter trap assay aggregates were spotted onto a cellulose acetate membrane (0.2 µm pore size, Whatman) in doublets. For antibody staining membranes were treated like Western blot membranes and were incubated with primary antibodies anti-HA (1:1000, Sigma- Aldrich, H3663), anti-ubiquitin (1:100, Sigma-Aldrich, U5379), anti-NEDD4 (1:500, Proteintech, 21698-1-AP) and anti-USP5 (1:500, Proteintech, 66213-1-lg), and subsequently incubated with secondary antibodies IRDye 680 or IRDye 800 (1:10,000; LI-COR Biosciences).

### ReAsH and FlAsH staining

Cells were transfected with the appropriate C4-tag containing plasmids. Cells were cultured on glass coverslips, rinsed with pre-warmed 1× PBS buffer and labeled for 30 min with pre-warmed DMEM containing 1 μM ReAsH or FlAsH (kindly provided by H. Overkleeft, University Leiden, The Netherlands) and 10 μM 1,2-ethanedithiol (EDT, Sigma-Aldrich) at 37 °C. After staining cells were washed twice with 1× PBS for subsequent analysis.

### Mouse models and tissue preparation

Brain sections from 14-week-old R6/2 mice^28^ and 22-month-old homozygous *Hdh*Q150 mice^29^ were prepared as described previously^49^. All experimental procedures performed on mice were conducted under a project license from the Home Office and approved by the King’s College London Ethical Review Process Committee in the UK, and are in accordance with relevant guidelines and regulations. Mice were anesthetized by i.p, injection of 100 µl sodium pentobarbital and perfused with 4% PFA in 0.1 M sodium phosphate buffer (NaH_2_PO_4_, pH 7.4). After dissecting the brains from the skull, brains were post-fixed in 4% PFA at 4 °C overnight and stored in 0.1 M NaH_2_PO_4_/0.5% PFA until further use. Brains were washed with PBS and incubated in 20% sucrose/PBS overnight. Subsequently, brains were frozen on dry ice and stored at −80 °C.

### Immunostaining of mouse tissue

Staining procedures are performed as described previously^49^. In short, coronally cut 10 µM thick sections of mouse brain cortex were post-fixed with 4% PFA for 15 min, subjected to antigen retrieval with 10 mM sodium citrate and 0.05% tween-20 at 85–95 °C for 10 min. Subsequently, sections were rinsed with PBS, blocked and permeabilized with 1% BSA, 2% FBS and 0.4% Triton X-100 in PBS for one hour. Brain sections were incubated overnight with primary antibodies against N-terminal Htt (S829, a kind gift of G, Bates King’s College, UK, 1:100)^30^ and anti-ubiquitin P4D1 (Santa Cruz, 1:100). Sections were subsequently washed and incubated with secondary antibodies anti-mouse Alexa488 or anti-goat Cy3 (1:700 Jackson ImmunoResearch Laboratories) for one hour. Finally, sections were embedded in Vectashield containing DAPI to stain nuclei (Vector Laboratories). All procedures were performed at room temperature.

### Immunostaining of cultured cells

One day prior transfection and/or electroporation cells were seeded on glass coverslips in a 35 mm^2^ dish. For antibody staining cells were fixed with 4% PFA and washed three times with PBS. Coverslips were blocked for 20 min with PBS containing 2% FBS, 1% BSA and 0.4% Triton-X100. After incubation with primary antibodies anti-HA (H3663, Sigma-Aldrich, 1:200) and anti-ubiquitin P4D1 (Santa Cruz, 1:100, sc-8017) for two hours, coverslips were washed three times with PBS and incubated with secondary antibody (anti-mouse-Alexa488, Jackson Laboratories) for one hour. Lastly, coverslips were washed three times with PBS and mounted on object glasses using ProlongGold mounting medium with DAPI (Life technologies, P36931). All procedures were performed at room temperature.

### Fluorescence microscopy and FRAP experiments

Images were taken using the Leica SP8-SMD confocal microscope with a 63× oil objective using UV (405 nm) and white light lasers (450–650 nm). For live cell imaging cells were seeded on glass coverslips in 35 mm^2^ plates and grown overnight as previously described. The next day cells were transfected with DNA constructs and stained with ReAsH or FlAsH, one hour prior to imaging. For FRAP (Fluorescence recovery after photobleaching) analysis on mHtt IBs, defined region of interest (ROI) of fluorescently-tagged Ub and ReAsH- or FlAsH-stained mHtt IBs were bleached at 100% laser power, and fluorescent recovery within this ROI was measured at 37 °C in time. Each FRAP analysis consisted of taking two pre-bleach images, five immediate post-bleach images every 0.863 seconds, followed by ten post-bleach images every 30 seconds and fifteen post-bleach images every 60 seconds in order to reduce the number of images needed.

For each FRAP experiment half of the circular aggregate was defined as ROI that was photobleached. For normalization an adjacent non-bleached area was used as a control for general photobleaching and background fluorescence at the corresponding time points. The values were normalized to pre-bleach and the first post-bleach was set to zero. For FRAP data analysis normalization between independent experiments was performed.

### Statistical analysis

All values were obtained from minimum three independent experiments and expressed as mean ± SD. Statistical analysis was performed using two-tailed Student’s *t*-test. *p* < 0.05 was considered statistically significant.

## Electronic supplementary material

Supplementary information

## References

[1] Ramdzan YM (2017). Huntingtin Inclusions Trigger Cellular Quiescence, Deactivate Apoptosis, and Lead to Delayed Necrosis. Cell reports.

[2] Schipper-Krom S (2014). Dynamic recruitment of active proteasomes into polyglutamine initiated inclusion bodies. FEBS letters.

[3] Raspe M (2009). Mimicking proteasomal release of polyglutamine peptides initiates aggregation and toxicity. Journal of cell science.

[4] DiFiglia M (1997). Aggregation of huntingtin in neuronal intranuclear inclusions and dystrophic neurites in brain. Science.

[5] Hipp MS (2012). Indirect inhibition of 26S proteasome activity in a cellular model of Huntington’s disease. The Journal of cell biology.

[6] Bennett EJ (2007). Global changes to the ubiquitin system in Huntington’s disease. Nature.

[7] Bersuker K, Brandeis M, Kopito RR (2016). Protein misfolding specifies recruitment to cytoplasmic inclusion bodies. The Journal of cell biology.

[8] Yu A (2014). Protein aggregation can inhibit clathrin-mediated endocytosis by chaperone competition. Proceedings of the National Academy of Sciences of the United States of America.

[9] Dantuma NP, Groothuis TA, Salomons FA, Neefjes J (2006). A dynamic ubiquitin equilibrium couples proteasomal activity to chromatin remodeling. The Journal of cell biology.

[10] Schipper-Krom S, Juenemann K, Reits EA (2012). The Ubiquitin-Proteasome System in Huntington’s Disease: Are Proteasomes Impaired, Initiators of Disease, or Coming to the Rescue?. Biochemistry research international.

[11] Gutekunst CA (1999). Nuclear and neuropil aggregates in Huntington’s disease: relationship to neuropathology. The Journal of neuroscience: the official journal of the Society for Neuroscience.

[12] Schilling G (2007). Characterization of huntingtin pathologic fragments in human Huntington disease, transgenic mice, and cell models. Journal of neuropathology and experimental neurology.

[13] Juenemann K (2011). Modulation of mutant huntingtin N-terminal cleavage and its effect on aggregation and cell death. Neurotoxicity research.

[14] Lunkes A (2002). Proteases acting on mutant huntingtin generate cleaved products that differentially build up cytoplasmic and nuclear inclusions. Molecular cell.

[15] Juenemann K, Wiemhoefer A, Reits EA (2015). Detection of ubiquitinated huntingtin species in intracellular aggregates. Frontiers in molecular neuroscience.

[16] Tsvetkov AS (2013). Proteostasis of polyglutamine varies among neurons and predicts neurodegeneration. Nature chemical biology.

[17] Juenemann K (2013). Expanded polyglutamine-containing N-terminal huntingtin fragments are entirely degraded by mammalian proteasomes. The Journal of biological chemistry.

[18] Hartl FU, Bracher A, Hayer-Hartl M (2011). Molecular chaperones in protein folding and proteostasis. Nature.

[19] Gong B, Kielar C, Morton AJ (2012). Temporal separation of aggregation and ubiquitination during early inclusion formation in transgenic mice carrying the Huntington’s disease mutation. PloS one.

[20] Skibinski GA, Boyd L (2012). Ubiquitination is involved in secondary growth, not initial formation of polyglutamine protein aggregates in C. elegans. BMC cell biology.

[21] Carlson N, Rechsteiner M (1987). Microinjection of ubiquitin: intracellular distribution and metabolism in HeLa cells maintained under normal physiological conditions. The Journal of cell biology.

[22] Kawaguchi Y (2003). The deacetylase HDAC6 regulates aggresome formation and cell viability in response to misfolded protein stress. Cell.

[23] Johnston JA, Ward CL, Kopito RR (1998). Aggresomes: a cellular response to misfolded proteins. The Journal of cell biology.

[24] El Oualid F (2010). Chemical synthesis of ubiquitin, ubiquitin-based probes, and diubiquitin. Angewandte Chemie.

[25] An H, Statsyuk AV (2015). An inhibitor of ubiquitin conjugation and aggresome formation. Chemical science.

[26] Steffan JS (2004). SUMO modification of Huntingtin and Huntington’s disease pathology. Science.

[27] Kim S, Nollen EA, Kitagawa K, Bindokas VP, Morimoto RI (2002). Polyglutamine protein aggregates are dynamic. Nature cell biology.

[28] Mangiarini L (1996). Exon 1 of the HD gene with an expanded CAG repeat is sufficient to cause a progressive neurological phenotype in transgenic mice. Cell.

[29] Lin CH (2001). Neurological abnormalities in a knock-in mouse model of Huntington’s disease. Human molecular genetics.

[30] Sathasivam K (2001). Centrosome disorganization in fibroblast cultures derived from R6/2 Huntington’s disease (HD) transgenic mice and HD patients. Human molecular genetics.

[31] Mulder MP (2016). A cascading activity-based probe sequentially targets E1-E2-E3 ubiquitin enzymes. Nature chemical biology.

[32] Chhangani D, Upadhyay A, Amanullah A, Joshi V, Mishra A (2014). Ubiquitin ligase ITCH recruitment suppresses the aggregation and cellular toxicity of cytoplasmic misfolded proteins. Scientific reports.

[33] Hao R (2013). Proteasomes activate aggresome disassembly and clearance by producing unanchored ubiquitin chains. Molecular cell.

[34] Tan JM (2008). Lysine 63-linked ubiquitination promotes the formation and autophagic clearance of protein inclusions associated with neurodegenerative diseases. Human molecular genetics.

[35] Stenoien DL, Mielke M, Mancini MA (2002). Intranuclear ataxin1 inclusions contain both fast- and slow-exchanging components. Nature cell biology.

[36] Dantuma NP, Bott LC (2014). The ubiquitin-proteasome system in neurodegenerative diseases: precipitating factor, yet part of the solution. Frontiers in molecular neuroscience.

[37] Gillis J (2013). The DNAJB6 and DNAJB8 protein chaperones prevent intracellular aggregation of polyglutamine peptides. The Journal of biological chemistry.

[38] Hjerpe R (2016). UBQLN2 Mediates Autophagy-Independent Protein Aggregate Clearance by the Proteasome. Cell.

[39] Li X (2010). Inhibiting the ubiquitin-proteasome system leads to preferential accumulation of toxic N-terminal mutant huntingtin fragments. Human molecular genetics.

[40] Bhat KP, Yan S, Wang CE, Li S, Li XJ (2014). Differential ubiquitination and degradation of huntingtin fragments modulated by ubiquitin-protein ligase E3A. Proceedings of the National Academy of Sciences of the United States of America.

[41] Mishra A (2008). E6-AP promotes misfolded polyglutamine proteins for proteasomal degradation and suppresses polyglutamine protein aggregation and toxicity. The Journal of biological chemistry.

[42] Zucchelli S (2011). Tumor necrosis factor receptor-associated factor 6 (TRAF6) associates with huntingtin protein and promotes its atypical ubiquitination to enhance aggregate formation. The Journal of biological chemistry.

[43] Tsai YC, Fishman PS, Thakor NV, Oyler GA (2003). Parkin facilitates the elimination of expanded polyglutamine proteins and leads to preservation of proteasome function. The Journal of biological chemistry.

[44] Iwata A (2009). Intranuclear degradation of polyglutamine aggregates by the ubiquitin-proteasome system. The Journal of biological chemistry.

[45] Cattaneo E, Conti L (1998). Generation and characterization of embryonic striatal conditionally immortalized ST14A cells. Journal of neuroscience research.

[46] An H, Statsyuk AV (2013). Development of activity-based probes for ubiquitin and ubiquitin-like protein signaling pathways. Journal of the American Chemical Society.

[47] Ekkebus R (2013). On terminal alkynes that can react with active-site cysteine nucleophiles in proteases. Journal of the American Chemical Society.

[48] de Jong A (2012). Ubiquitin-based probes prepared by total synthesis to profile the activity of deubiquitinating enzymes. Chembiochem: a European journal of chemical biology.

[49] Jansen AH (2017). Frequency of nuclear mutant huntingtin inclusion formation in neurons and glia is cell-type-specific. Glia.

