# Supplementary material for: Dynamic recruitment of ubiquitin to mutant huntingtin inclusion bodies
Source: Sci Rep. 2018 Jan 23;8:1405. doi: 10.1038/s41598-018-19538-0 (PMC5780509; doi:10.1038/s41598-018-19538-0)
Supplement: Supplementary file 1 — Supplementary information [file 41598_2018_19538_MOESM1_ESM.docx]

**Dynamic recruitment of ubiquitin to mutant huntingtin inclusion bodies**

Katrin Juenemann^1,2^, Anne H.P. Jansen^1^, Luigi van Riel^1^, Remco Merkx^3^, Monique P.C. Mulder^3,4^, Heeseon An^5,6^, Alexander Statsyuk^6^, Janine Kirstein^2^, Huib Ovaa^3,4^, Eric A. Reits^1^*

**Supplementary methods**

***In vitro* ubiquitination assay**

E6AP-mediated ubiquitination of the protein S5a was performed according to the manufacturer’s instructions (BostonBiochem, Cat. #K230). Synthesized TAMRA-Ub was used with an end concentration of 200 ng/µl. The reaction was terminated by addition of 5x Loading buffer and 1 µl 1M DTT. Ubiquitination of the protein S5a was detected by the anti-S5a antibody (BostonBiochem, AF5540).

**Supplementary figure 1**

**
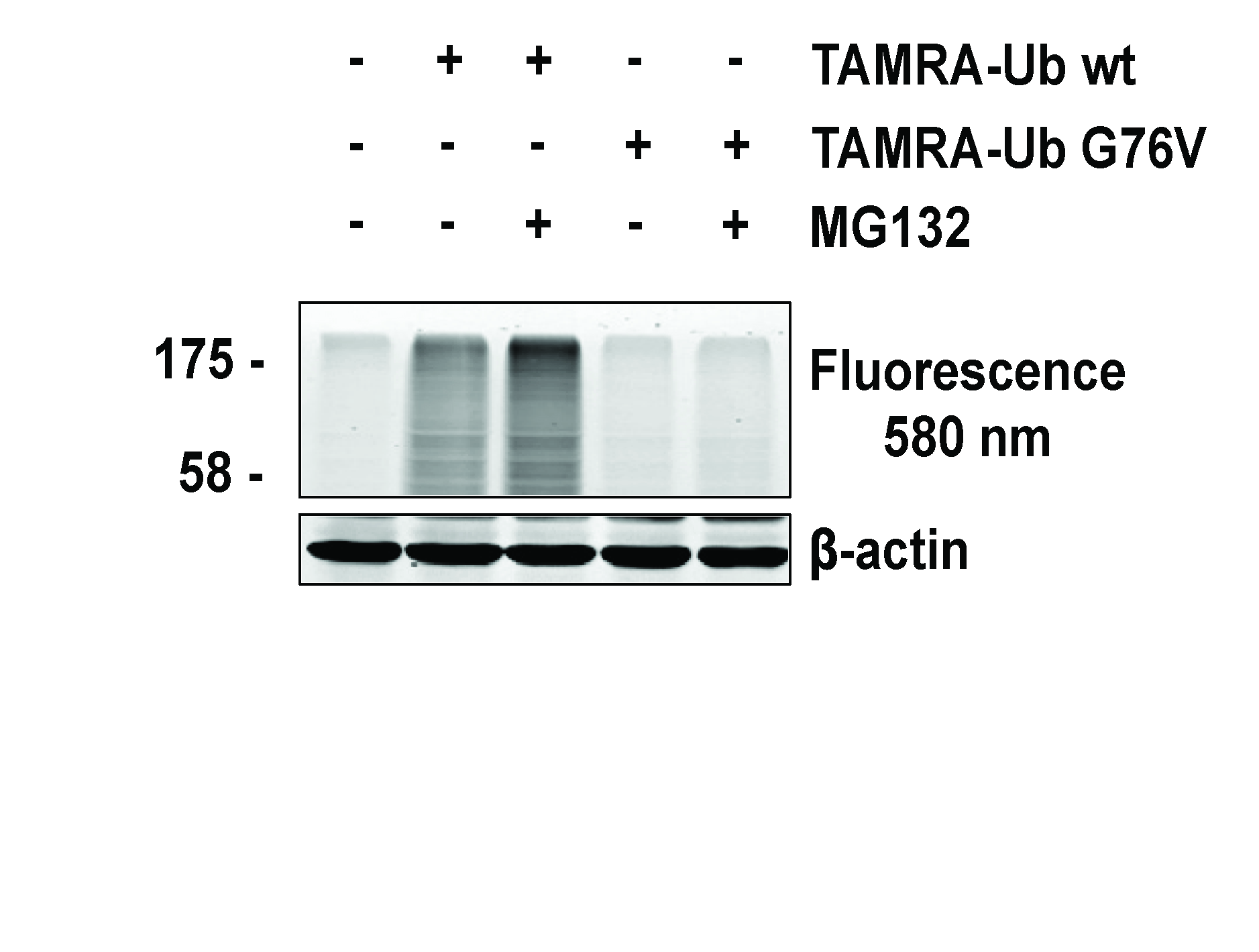
**

**Supplementary figure 1S:** TAMRA-Ub incorporation into endogenous polyUb chains. Fluorescence scan of SDS-PAGE loaded with cell lysates of Neuro-2A cells electroporated with TAMRA-Ub wildtype (wt), mutant

TAMRA-Ub G76V and non-electroporated cells, respectively. One hour after electroporation cells were treated with 20 µM MG132 for additional two hours. β-actin was used as a loading control.

**Supplementary figure 2**


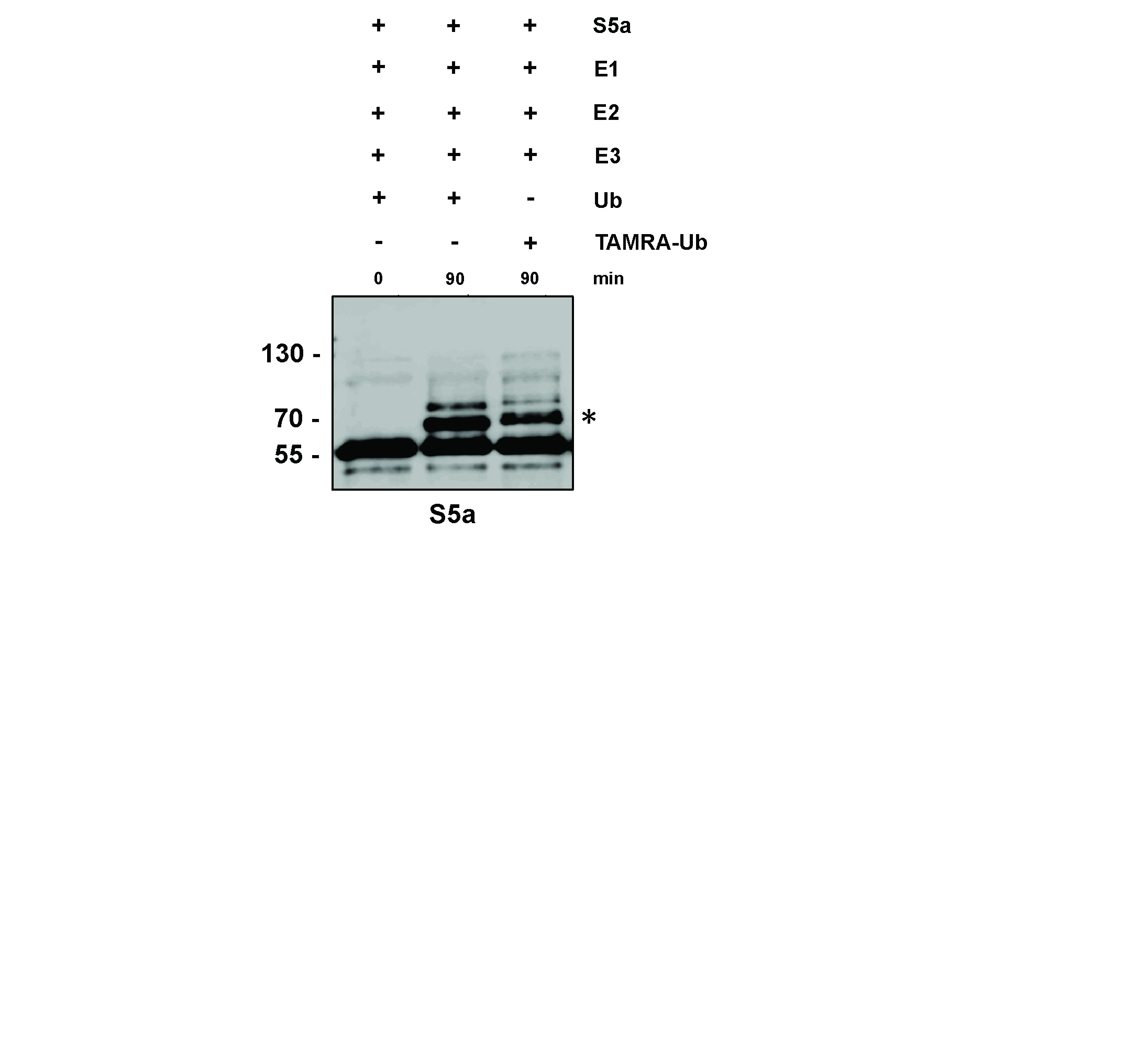


**Supplementary figure 2S:** TAMRA-Ub conjugation to the protein S5a.

*In vitro* ubiquitination of the protein S5a within 90 min. Mono-ubiquitination of the S5a protein (asterisk) by Ub and TAMRA-Ub shown by the anti-S5a antibody.

**Supplementary figure 3**

**
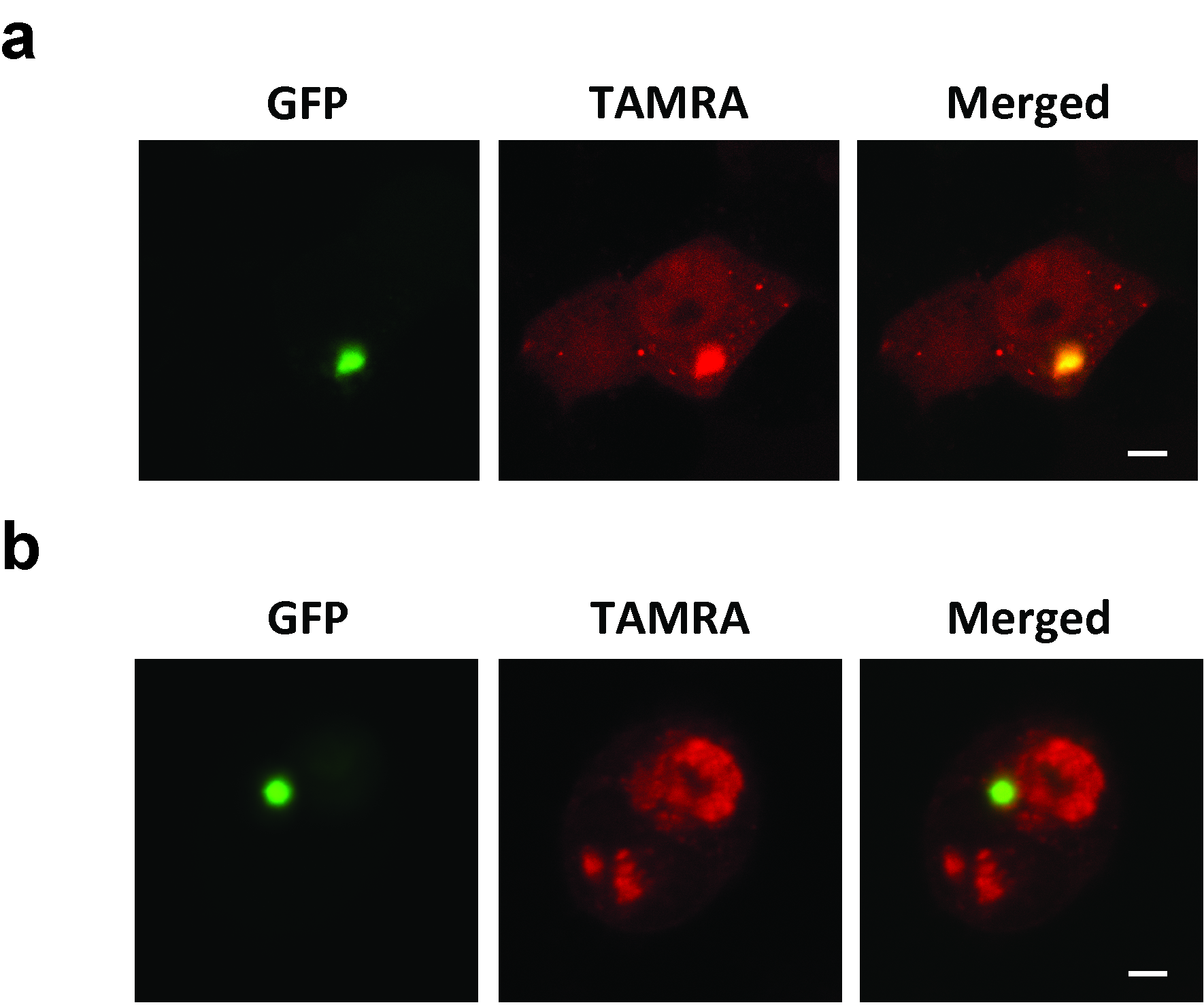
**

**Supplementary figure 3S:** TAMRA-Ub distribution in cells with mHtt IBs.

**a)** Confocal images of Neuro-2A cells transfected with Htt-exon1-97Q-GFP. 24 hours after transfection cells were electroporated with TAMRA-Ub and incubated for additional 24 hours. Following a second electroporation of TAMRA-Ub cells were visualized after two hours by confocal microscopy. Scale bar: 3 µm. **b)** Confocal images of Neuro-2A cells transfected with

Htt-exon1-97Q-GFP. 24 hours after transfection cells were electroporated with TAMRA-Ub. One hour after electroporation cells were treated with 50 µM epoxomicin for additional 24 hours. Scale bar: 3 µm.

**Supplementary figure 4**

**
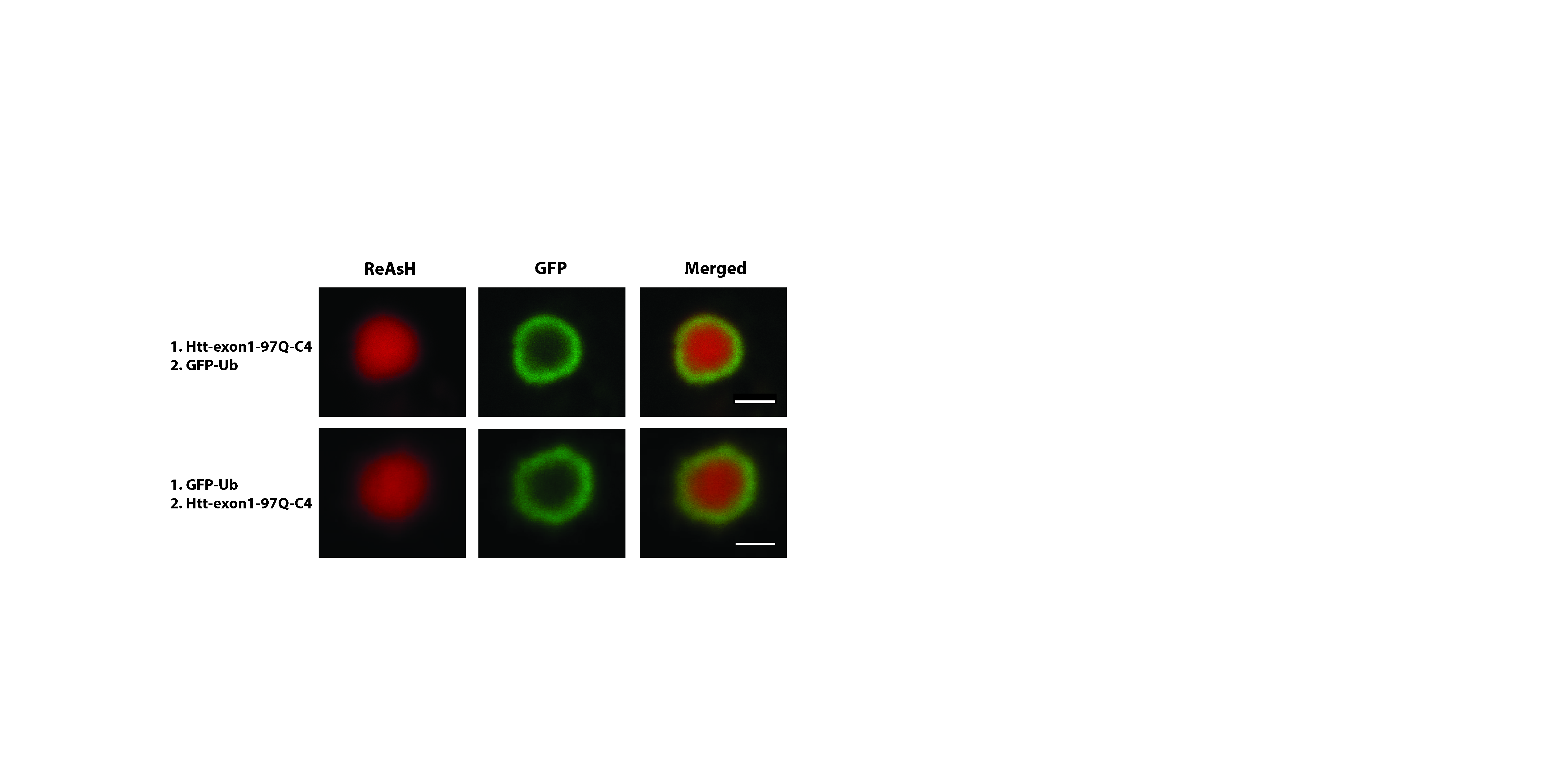
**

**Supplementary figure 4S:** Sequential expression of GFP-Ub and Htt-exon1-97Q-C4 exhibit GFP-Ub ring-like structure at IBs. Confocal images of

Neuro-2A cells transfected with GFP-Ub and 24 hours later transfected with Htt-exon1-97Q-C4, and vice versa. 24 hours later cells were ReAsH-stained and fixed for imaging. Scale bar: 2 µm.

**Supplementary figure 5**


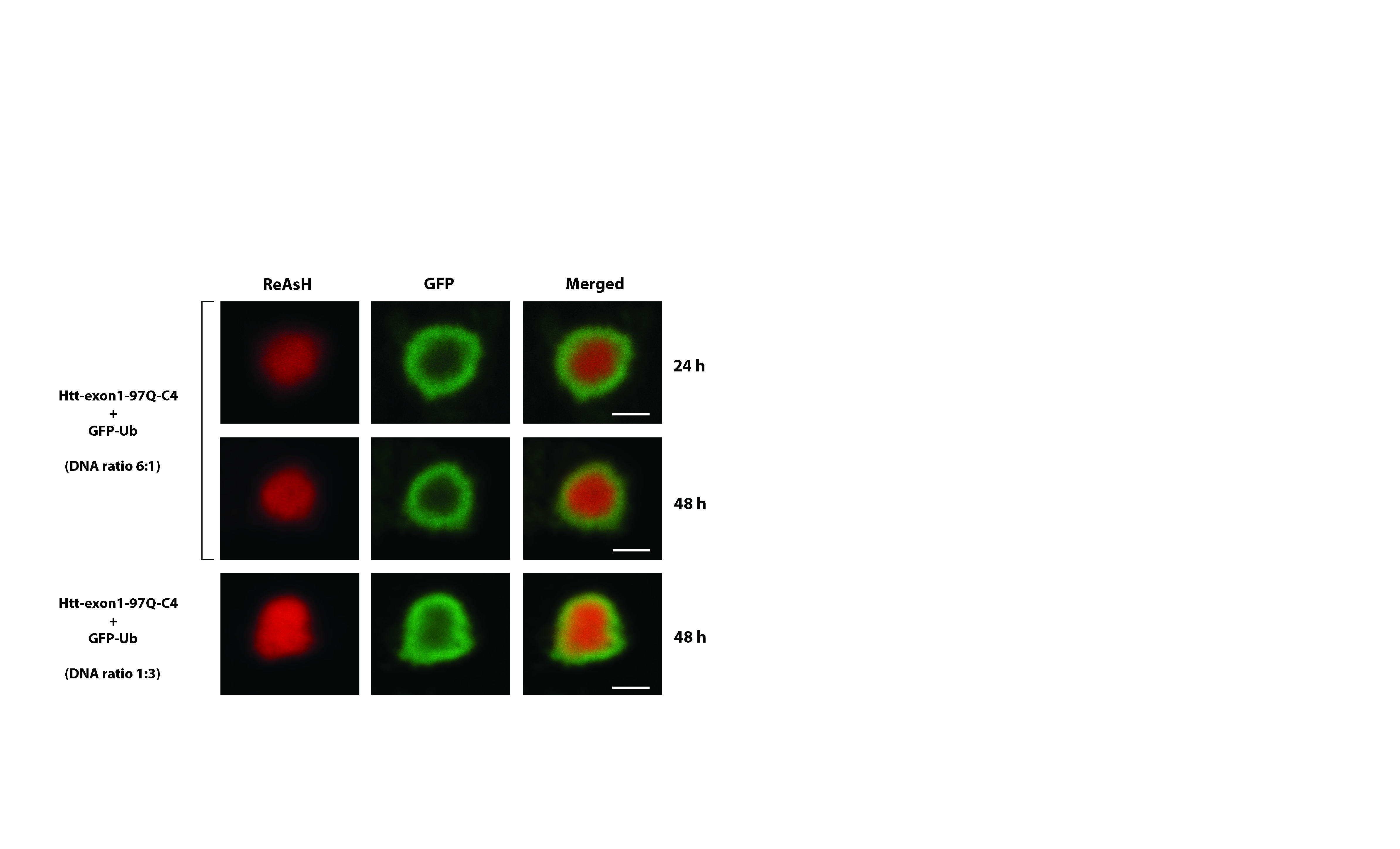


**Supplementary figure 5S:** Co-expression of GFP-Ub and Htt-exon1-97Q-C4 exhibit GFP-Ub ring-like structure at IBs. Confocal images of Neuro-2A cells co-transfected with different plasmid ratios of GFP-Ub and Htt-exon1-97Q-C4. 24 and 48 hours later cells were ReAsH-stained and fixed for imaging. Scale bar: 2 µm.
